# Cytosolic Glutamine Synthetase GS1;3 Is Involved in Rice Grain Ripening and Germination

**DOI:** 10.3389/fpls.2022.835835

**Published:** 2022-02-08

**Authors:** Takayuki Fujita, Marcel Pascal Beier, Mayumi Tabuchi-Kobayashi, Yoshitaka Hayatsu, Haruka Nakamura, Toshiko Umetsu-Ohashi, Kazuhiro Sasaki, Keiki Ishiyama, Emiko Murozuka, Mikiko Kojima, Hitoshi Sakakibara, Yuki Sawa, Akio Miyao, Toshihiko Hayakawa, Tomoyuki Yamaya, Soichi Kojima

**Affiliations:** ^1^Graduate School of Agricultural Science, Tohoku University, Sendai, Japan; ^2^Faculty of Science/Institute for the Advancement of Higher Education, Hokkaido University, Sapporo, Japan; ^3^Graduate School of Agricultural and Life Sciences, The University of Tokyo, Tokyo, Japan; ^4^Center for Sustainable Resource Science, RIKEN, Yokohama, Japan; ^5^Graduate School of Bioagricultural Sciences, Nagoya University, Nagoya, Japan; ^6^Institute of Crop Science, National Agriculture and Food Research Organization, Tsukuba, Japan; ^7^Division for Interdisciplinary Advanced Research and Education, Tohoku University, Sendai, Japan

**Keywords:** rice, GS1, yield, nitrogen translocation, germination, amino acids, grain filling

## Abstract

Ammonium is combined with glutamate to form glutamine. This reaction is catalyzed by glutamine synthetase (GS or GLN). Plants harbor several isoforms of cytosolic GS (GS1). Rice *GS1;3* is highly expressed in seeds during grain filling and germination, suggesting a unique role in these processes. This study aimed to investigate the role of GS1;3 for rice growth and yield. *Tos17* insertion lines for *GS1;3* were isolated, and the nitrogen (N), amino acid, and ammonium contents of *GS1;3* mutant grains were compared to wild-type grains. The spatiotemporal expression of *GS1;3* and the growth and yield of rice plants were evaluated in hydroponic culture and the paddy field. Additionally, the stable isotope of N was used to trace the foliar N flux during grain filling. Results showed that the loss of GS1;3 retarded seed germination. Seeds of *GS1;3* mutants accumulated glutamate but did not show a marked change in the level of phytohormones. The expression of *GS1;3* was detected at the beginning of germination, with limited promoter activity in seeds. *GS1;3* mutants showed a considerably decreased ripening ratio and decreased N efflux in the 12th leaf blade under N deficient conditions. The *β-glucuronidase* gene expression under control of the *GS1;3* promoter was detected in the vascular tissue and aleurone cell layer of developing grains. These data suggest unique physiological roles of GS1;3 in the early stage of seed germination and grain filling under N deficient conditions in rice.

## Introduction

Nitrogen (N) is one of the most important macronutrients required for plant growth (Marschner, 1995). Plants absorb inorganic N from the soil in the forms of free N ions, nitrate, and ammonium (Marschner, 1995). Glutamine synthetase (GS or GLN) combines ammonium with glutamate in an ATP-dependent manner to form glutamine (Lea and Azevedo, 2007; Thomsen et al., 2014). Two types of GS enzymes are present in plants: cytosolic GS (GS1) and chloroplastic GS (GS2). Quantitative trait locus (QTL) analyses suggested the importance of GS1 for N use efficiency in crop plants (Obara et al., 2001; Gallais and Hirel, 2004; Habash et al., 2007; Gadaleta et al., 2014; Thomsen et al., 2014). Plants harbor several *GS1* genes (Orsel et al., 2014), for example, the rice (*Oryza sativa*) genome encodes three GS1 enzymes (Tabuchi et al., 2005). Variable spatiotemporal distribution of *GS1* expression and different enzymatic characteristics of GS1 isozymes suggests that each GS1 isozyme has a specific physiological function (Thomsen et al., 2014).

Some of the GS1 isoforms exhibit seed-specific expression. The contribution of these seed-specific GS1 isoforms toward the growth and productivity of crop plants remains unclear, although several lines of evidence indicate the importance of GS1 in cereal grains. For example, in rice, *GS1;1* is expressed in the vascular tissue of ripening grains (Yabuki et al., 2017) and GS1;3 is highly expressed in both maturing and germinating grains (Tabuchi et al., 2005, 2007). In barley (*Hordeum vulgare*), all GS1 isoforms are detected in barley grains during the early milk stage (Goodall et al., 2013).

In *Arabidopsis thaliana*, the involvement of GS1 isozymes, Gln1;1 and Gln1;2, in the germination and production of seeds has been demonstrated using reverse genetics (Guan et al., 2015) though both Gln1 isoforms are not seed-specific (Lothier et al., 2011; Guan et al., 2015, 2016; Guan and Schjoerring, 2016; Konishi et al., 2017, 2018). Microarray analysis has shown that Arabidopsis *GLN1;5* is highly expressed in ripening seeds (Schmid et al., 2005; Le et al., 2010). The expression of *GLN1;5* is also observed in germinating seeds (Bassel et al., 2008). These data reveal a unique expression profile of *GLN1;5* during the life cycle of Arabidopsis plants.

However, reverse genetics studies directly linking the seed-specific expression of *GS1* genes with crop productivity are limited because of the unavailability of knockout mutants. Rice GS1;1 is ubiquitously expressed in whole plants and is essential for normal growth, as the loss of GS1;1 leads to severe growth retardation (Tabuchi et al., 2005; Kusano et al., 2011). In maize (*Zea mays*), Gln1-3 and Gln1-4 are expressed in leaves and are involved in grain production, which suggests their importance during N translocation to source organs (Martin et al., 2006). Rice GS1;2 is expressed mainly in roots and is thought to be responsible for primary ammonium assimilation in roots, as GS1;2 knockout mutants accumulate free ammonium in xylem sap under high ammonium supply (Funayama et al., 2013).

In this study, we investigated the role of GS1;3, a seed-specific isoform, during seed germination and grain filling in rice. Real-time PCR and promoter analyses revealed the temporal and spatial distribution of *GS1;3* expression in rice. We also isolated and characterized *GS1;3* knockout rice mutants. We measured the concentrations of amino acids and plant hormones in germinating seeds, tissue dry weight, and N flux in leaves using a stable isotope tracer during reproductive growth of *GS1;3* knockout mutants. Our results suggest that rice GS1;3 functions in ammonium assimilation in the aleurone layer and the endosperm during storage protein biosynthesis and degradation, as well as it is likely involved in the energy provision for these processes. GS1;3 is therefore important for seed germination and spikelet filling, with a more pronounced function during nitrogen limitation.

## Materials and Methods

### Seed Material

All seeds used in this study derived from rice plants cv. Nipponbare, harvested on the 19th October 2010 in the Kashimadai Field of the Tohoku University in Japan. Before experimental use, seed dormancy was broken by incubation at 30°C for 7 days. Seeds were selected by water density (1.13 g ml^−1^), incubated at 60°C for 10 min, and sterilized by 70% ethanol for 30 s followed by 2% of sodium hypochlorite for 20 min and subsequent washing. The following steps were described in the respective protocols.

### Definition of Seed and Plant Stages, Germination, and Yield Component Analysis

Filled spikelet, selected by saltwater with a density of 1.06 g ml^−1^, is the term used for seeds in the yield component analysis, while mature seeds, selected by saltwater with a density of 1.13 g ml^−1^ were used for physiological analysis.

Developing seeds are harvested before they reach full maturity. During the rice ripening stage, 5 sub-stages can be defined as heading, milky, dough, yellow ripe, and maturity stage. The specific sub-stages were therefore either dough or yellow ripe (before the seeds turn brown on the surface).

A seed was regarded as germinating when the radicle emerged from the husk.

The general life cycle of rice plants after seedling establishment can be divided into vegetative (until tiller reaches maximal height), the reproductive stage (until heading), and finally the ripening stage (until harvest).

The yield component analysis was done to determine which of the main determinants of yield, the panicle number, the weight of brown rice (dehusked filled spikelets), the spikelet number, and the ratio of filled spikelets compared to unfilled spikelets at harvest (not sinking in saltwater with a density of 1.06 g ml^−1^) is influenced in the mutants compared to the WT.

All definitions are based on Yoshida (1981).

### Hydroponic Culture

Mature seeds of WT Nipponbare and *GS1;3* mutants were germinated on a moistened filter paper in Petri dishes at 30°C for 2 days. The seeds were placed on nets floating on 8 l of water with a pH adjusted to 5.8 by HCl. Twenty three days after sowing (DAS), single seedlings were rolled in moltopren and fixed to holes in a sieve placed on a Wagner pot (1:5000). Each Wagner pot contained 4 seedlings.

The nutrient solution used from this point on was based on a previous description (Mae and Ohira, 1981), with slight modifications: 0.5 mM NH_4_Cl, 0.6 mM NaH_2_PO_4_, 0.3 mM K_2_SO_4_, 0.3 mM CaCl_2_, 0.6 mM MgCl_2_, 45 μM Fe-EDTA, 50 μM H_3_BO_3_, 9 μM MnSO_4_, 0.3 μM CuSO_4_, 0.7 μM ZnSO_4_, and 0.1 μM Na_2_MoO_4_. The nutrient solution was buffered at pH 5.5.

The strength of the nutrient solution was changed over time, and the solution was renewed every 4–6 days. One-quarter strength solution was used for 13 days (until 35 DAS), followed by half-strength solution for 11 days (until 46 DAS) and full-strength solution for 61 days (until 107 DAS). With the start of heading 107 DAS, the media was changed to one-quarter strength solution for 20 days (until 127 DAS) and to one-eighth strength solution for 5 days (until 132 DAS), followed by water until harvest.

### Field Experiment

Plants of WT Nipponbare and *Tos17* insertion mutant lines of GS1;3 (*gs1;3-1* and *gs1;3-2*) were grown in a paddy field in Kashimadai, Miyagi, Japan for measuring agronomical traits. Seeds were sown in cell trays, with one seed per cell, and grown in the greenhouse under natural light for 29 days. Seedlings were then transplanted in the paddy field. The paddy field was fertilized with 30 kg ha^−1^ of basal fertilizer containing 16% each of N (4.8 kg ha^−1^of N), P, and K (Coop Chemical Co., Tokyo, Japan) and additionally with 27 kg ha^−1^of N (5.7 kg ha^−1^of N) fertilizer in the form of ammonium sulfate (Ube Material Industries, Ube, Japan), before transplanting, resulting in a total N supply of 10.5 kg ha^−1^. At 153 days after germination, five plants were harvested at the soil surface and used for measuring the panicle number, shoot dry weight, and panicle dry weight. Grain and straw were dried in a greenhouse and then in a dry chamber.

### Yield Analysis

Brown rice yield was determined by multiplication of (1) panicle number per individual plant, (2) spikelet number per panicle, (3) filled spikelet ratio, and (4) weight of brown rice. Panicle number was counted from five plants after harvest. The number of spikelets was determined by counting all spikelets after the separation from the panicle. Filled spikelets were determined as spikelets that sank in saltwater (density at 1.06 g ml^−1^) and unfilled spikelets as floated. The ratio of filled and unfilled spikelets was defined as filled spikelets (%). Filled spikelets were washed in tap water and dried. The weight of brown rice was determined after the harvest of brown rice by husking of filled spikelets.

### Primers

All primers used in this article are listed in Supplementary Table S1.

### Isolation of *Tos17* Insertion Lines of GS1;3

*Tos17* insertion lines of *GS1;3* (Os03g0712800) were identified through the mutant panel database.^1^ For the selection of not-listed *Tos17* insertion lines, a nested PCR-based screening (Funayama et al., 2013) was carried out with pooled DNA samples provided by the National Institute of Agrobiological Sciences, NIAS, Tsukuba, Japan (Miyao et al., 2003). *Tos17* and *GS1;3* specific primers were used for the initial as well as the subsequent (nested) PCR. For the reactions, which were all conducted using the Gene Amp PCR System 9,700 (Thermo Fisher Scientific KK, Yokohama, Japan), the LA Taq DNA polymerase (Takara Bio Inc., Shiga, Japan) was used. Nested PCR products were purified and sequenced.

Seeds containing an identified *Tos17* insertion in *GS1;3* were obtained from NIAS.

Genotyping of the two candidate lines ND0163, and NE4721 with genomic DNA as template was conducted as previously described (Tabuchi et al., 2005). Homozygous lines of ND0163 were named *gs1;3-1*, and NE4721 were named *gs1;3-2*.

### Quantitative Real-Time PCR

Total RNA from mature seeds and seedlings was extracted with benzyl chloride (Suzuki et al., 2004) or the RNeasy Plant Mini Kit (Qiagen, Hilden, Germany), respectively. The RNA quality was determined using the Agilent 2,100 Bioanalyzer (Agilent Technologies Japan Ltd., Tokyo, Japan). Reverse transcription was carried out using either the SuperScript First-Strand Synthesis System for RT-PCR (Invitrogen, Tokyo, Japan), with 1 μg of total RNA, or the PrimeScript RT reagent Kit with gDNA Eraser (Takara Bio Inc., Otsu, Shiga, Japan), with 0.5 μg of total RNA, according to the manufacturer’s instructions. The resulting cDNA was used to analyze the expression of *OsGS1;1* (Ishiyama et al., 2004), *OsGS1;2*, *OsGS1;3* (Tabuchi et al., 2005), and *actin* (Sonoda et al., 2003) with qPCR.

The qPCR was conducted in Light Cycler Capillaries (Roche Diagnostics K.K., Tokyo, Japan) using an initial denaturation for 10 min at 95°C, followed by 40 cycles of 95°C for 10 s, 60°C for 10 s, and 72°C for 7 s. Each 20 μl reaction contained 1 μl cDNA, 1x LC FastStart DNA Master SYBR Green I (Roche), and 0.5 μM primers. A standard curve was generated from a serial dilution of cDNA. The expression of GS1 genes was normalized relative to actin. Results were shown as a mean value of three independent samples harvested from three individual plants.

### Semi-quantitative RT-PCR

Total RNA from mature not imbibed seeds was extracted with Sepasol RNA I Super G (Nacalai Tesque, Kyoto, Japan) according to the manufacturer’s instructions. The reverse transcription was carried out with the Prime Script RT reagent Kit with gDNA Eraser, as described for the qPCR. Gene-specific primers for *OsGS1;1* (Ishiyama et al., 2004), *OsGS1;2*, *OsGS1;3* (Tabuchi et al., 2005), and actin (Sonoda et al., 2003) were used for the semi-quantitative RT-PCR. The reactions were conducted in a Takara PCR thermal cycler with either 30 or 35 cycles of 98°C for 10 s, 60°C for 30 s, and 72°C for 30 s, and a final elongation for 1 min. Each reaction contained 0.5 U Takara Ex Taq, 1x Ex Taq Buffer, 4 nmol dNTPs, 5 ng cDNA, and 2 pmol primers. The semi-quantitative RT-PCR products were separated on a 1.5% agarose gel and stained with ethidium bromide.

### Vector Construction and Plant Transformation

As a promoter, 1983 bp of the *GS1;3* upstream region were amplified from the genomic DNA of *Oryza sativa L. ssp. Japonica* cv. Nipponbare and flanked with *attB* sites in a subsequent PCR (Hartley et al., 2000). The promoter sequence was cloned into pDONR221 *via* BP clonase (Thermo Fisher Scientific KK, Yokohama, Japan). Following sequence confirmation and linearization with PvuII and XhoI, the promoter was transferred from pDONR221 to pGWB3 (Nakagawa et al., 2007) *via* LR clonase (Thermo Fisher Scientific KK), resulting in the final construct *proGS1;3::GUS*. The recombinant vector was used for Agrobacterium-mediated transformation of rice as described previously (Hiei et al., 1994), with slight modifications (Kojima et al., 2000). Genomic DNA isolated from transgenic lines was subjected to PCR analysis to confirm the transformation.

### GUS Staining

Sterilized seeds were incubated in water at 4°C for 5 days in the dark for seed synchronization and then transferred to 30°C in the dark and incubated for either 0 h or 72 h. Seedlings were transferred to a 20 l container containing 14 kg of silt loam soil and 24 g of slow fertilizer containing 16% each of N, P, and K (Coop Chemical Co., Tokyo, Japan). Plants were cultured in a greenhouse with 26°C during a 14 h light period with supplementary artificial light, and 23°C during a 10 h dark period. Developing seeds were harvested at 35 days after flowering. For the GUS staining procedure seeds at both germination stage and ripening stage were cut in half and incubated in the GUS staining solution (100 mM sodium phosphate [pH 7.0], 10 mM EDTA, 0.5 mM K_3_Fe(CN)_4_, 0.5 mM K_4_Fe(CN)_6_-3H_2_O, 0.1% Triton X, 1 g L^−1^ X-Gluc, and 20% methanol; Kojima et al., 2000). Images were taken with a stereomicroscope (MZ12.5, Leica Microsystems K. K., Tokyo, Japan).

### Germination Ratio Measurement

The mature seed germination ratio for WT Nipponbare, *GS1;3* and *GS1;2* insertion lines (Funayama et al., 2013) was analyzed as previously described (Sasaki et al., 2015). Fully ripened seeds were germinated in 9 cm diameter Petri dishes containing 4.5 ml water. Petri dishes were kept in a dark incubator box at 30°C for 72 h. Four independent repetitions, each containing 50 seeds per line, were tested. The ratio of germinated seeds was determined 14 times during 72 h for each independent set-up.

### Amino Acid Measurement

Mature seeds of WT Nipponbare were imbibed in water for 0, 12, 24, 48, or 72 h. Another set of WT, *gs1;3-1*, and *gs1;3-2* seeds were imbibed in water for 0, 30, or 66 h. After imbibition, seeds were frozen in liquid N and powdered with an MB601U Multi-Bead Shocker (Yasui Co. Ltd., Tokyo, Japan). A 10-fold volume of 10 mM HCl was added, and the samples were homogenized with the Multi-Bead Shocker. The samples were cleared from debris by a 21,900 · *g* centrifugation for 10 min, and the supernatant was filtered with an Amicon Ultra-0.5 Centrifugal Filter (Millipore, Tokyo, Japan). Samples were derivatized with the AccQ-Fluor Reagent Kit (Nihon Waters K. K., Tokyo, Japan), and amino acid concentrations were determined as previously described (Tabuchi et al., 2005). Three to five independent samples were used for each data point, the samples were harvested from individual plants.

### Phytohormone Measurement

Mature seeds of WT Nipponbare, *gs1;3-1*, and *gs1;3-2* were imbibed in water for 24 h. After imbibition, seeds were frozen in liquid N and powdered with MB601U Multi-Bead Shocker (Yasui Co. Ltd., Tokyo, Japan). Concentrations of plant hormones were determined as previously described (Kojima et al., 2009). The plant hormone cytokinin was quantified *via* ultra-performance liquid chromatography (UPLC)-electrospray interface (ESI) tandem quadrupole mass spectrometry (qMS/MS; AQUITY UPLC™ System/Xevo-TQS; Waters) as described previously (Kojima et al., 2009). Auxins, gibberellins, abscisic acid, salicylic acid, and jasmonic acid were quantified with ultra-high-performance liquid chromatography (UHPLC)-ESI quadrupole-orbitrap mass spectrometer (UHPLC/Q-Exactive™; Thermo Scientific) as described previously (Kojima and Sakakibara, 2012; Shinozaki et al., 2015). Three to four independent samples were used for each data point.

### ^15^N Tracing and N Content

When the 12th leaf was expanding, plants were supplied with ^15^N-labeled 0.5 mM NH_4_Cl (4.06 atom %) for 5 days. Leaves from the 8th leaf to the flag leaf, main stem panicles, and leaf blades, leaf sheaths, and panicles of tillers were harvested. Dry weights of the 8th, 9th, 10th, 11th, 12th, 13th, and 14th leaf blades and main stem panicles were measured. Four plants were used for the measurements per genotype. Yield components were determined as previously described (Tamura et al., 2010). The 12th leaf blades were powdered with Multi-Beads Shocker, and the ^15^N/^14^N ratio and N content were determined with an elemental analyzer (Flash 2000, Thermo Fisher Scientific, Tokyo, Japan) equipped with isotope ratio MS (Delta V Advantage, Thermo Fisher Scientific). Efflux and influx were calculated as previously described (Makino et al., 1984).

### Statistical Analysis

Data were analyzed using Microsoft Excel add in software (Social Survey Research Information Co., Ltd., Tokyo, Japan). Correlation and partial correlation coefficients were determined between the root system architecture and biomass of plants. Correlations with a Value of *p* < 0.05 were considered statistically significant.

## Results

### *GS1;3* Is Highly Expressed in the Aleurone Layer and Endosperm During Seed Germination

**Figure 1:**
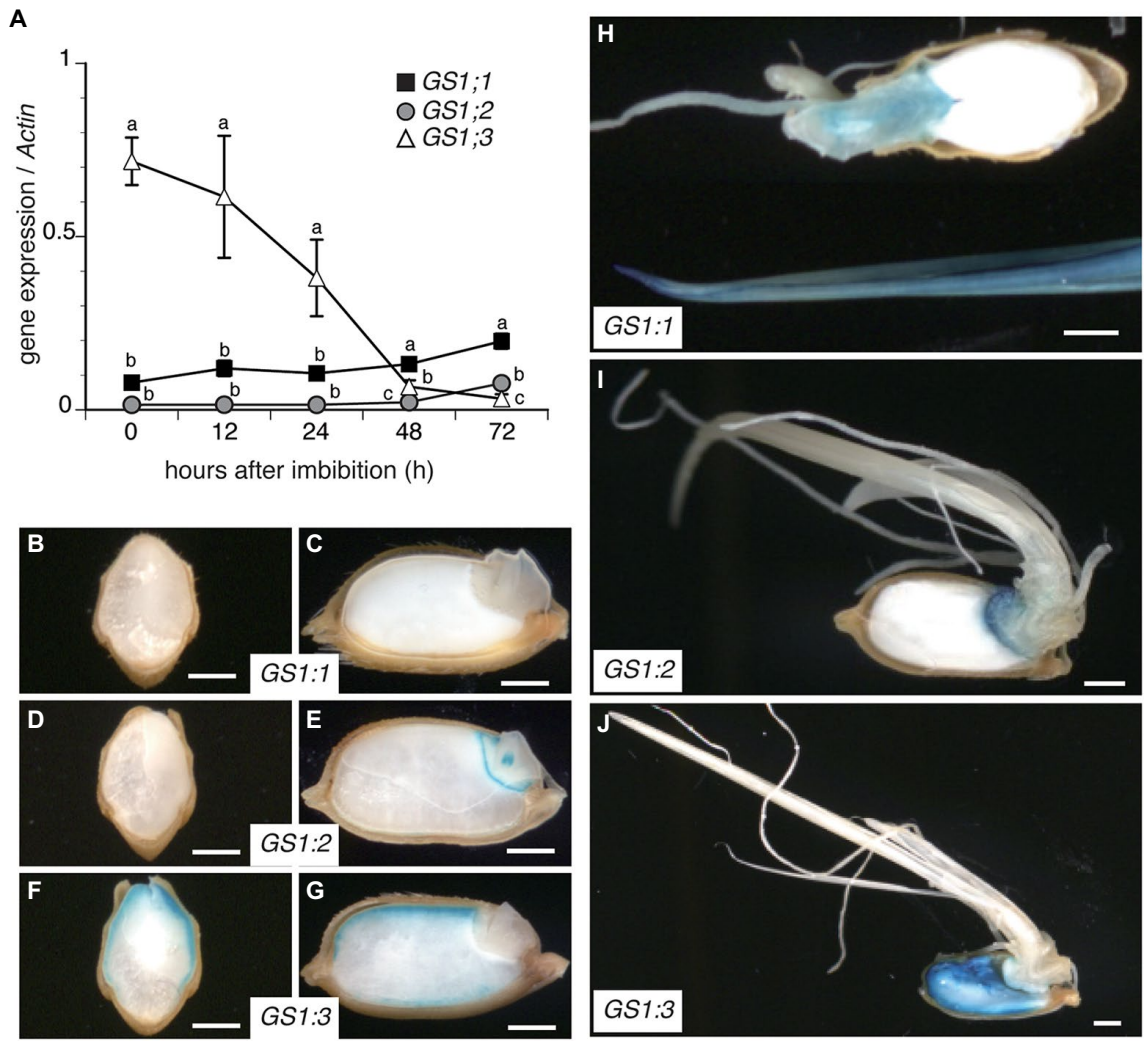
Differential expression and localization of cytosolic glutamine synthetase (GS1) genes during rice seed germination. **(A)** Time course qPCR analysis of *GS1* isoforms in WT plants during rice seed germination after imbibition of mature seeds. Data represent mean ± SD (*n* = 3). **(B–J)** *GS1* promoter-driven GUS activity in germinating rice seeds. Transgenic rice seeds harboring *GS1;1pro::GUS*, *GS1;2pro::GUS*, or *GS1;3pro::GUS* construct were germinated and cultured under controlled conditions for 72 h. Transverse **(B,D,F)** and longitudinal **(C,E,G)** sections of seeds expressing *GS1;1* **(B,C,H)**, *GS1;2* **(D,E,I)**, and *GS1;3* **(F,G,J)**. Images of seeds and seedlings were captured using a stereomicroscope at 24 h **(B–G)** and 72 h **(H–J)** after imbibition. Bars indicate 1 mm.

To investigate the temporal expression of GS1 isoforms during rice seed germination, a quantitative PCR (qPCR) analysis was conducted on wild-type (WT) Nipponbare seeds in a 72 h time course after imbibition (Figure 1A). While the expression of *GS1;3* was the highest at imbibition and decreased over time, *GS1;1* and *GS1;2* expression levels were low in the beginning of germination. From 48 h after imbibition onwards, *GS1;1* had the highest expression of all 3 isoforms, whereas *GS1;2* exceeded *GS1;3* expression at 72 h (Figure 1A).

Besides the temporal analysis by qPCR, the spatial expression of the GS1 isoforms was analyzed by β-glucuronidase (GUS) expression under the control of *GS1* isoform promoters.

At 24 h after imbibition, no GUS activity was detected in *proGS1;1::GUS* transgenic lines (Figures 1B,C), whereas *proGS1;2::GUS* was detected in the embryo (Figures 1D,E). The *proGS1;3::GUS* line showed an expression in the aleurone layer (Figures 1F,G). To visualize the expression pattern after the emergence of roots and shoots, the same transgenic lines were observed 72 h after imbibition (Figures 1H–J). The *GS1;1* promoter showed an expression in the shoots and roots (Figure 1H), while the *GS1;2* promoter showed mainly expression in the embryo with only a slight expression in the roots and shoots (Figure 1I). In contrast, the *GS1;3* promoter line showed an additional expression in the endosperm, compared to the aleurone-specific expression detected at 24 h after imbibition (Figure 1J).

These data suggest a physiological role of GS1;3 in the aleurone and endosperm during seed germination, a physiological role of GS1;2 in the embryo and a general function of GS1;1 in roots and shoots.

### Loss of GS1;3 Delays Seed Germination

**Figure 2:**
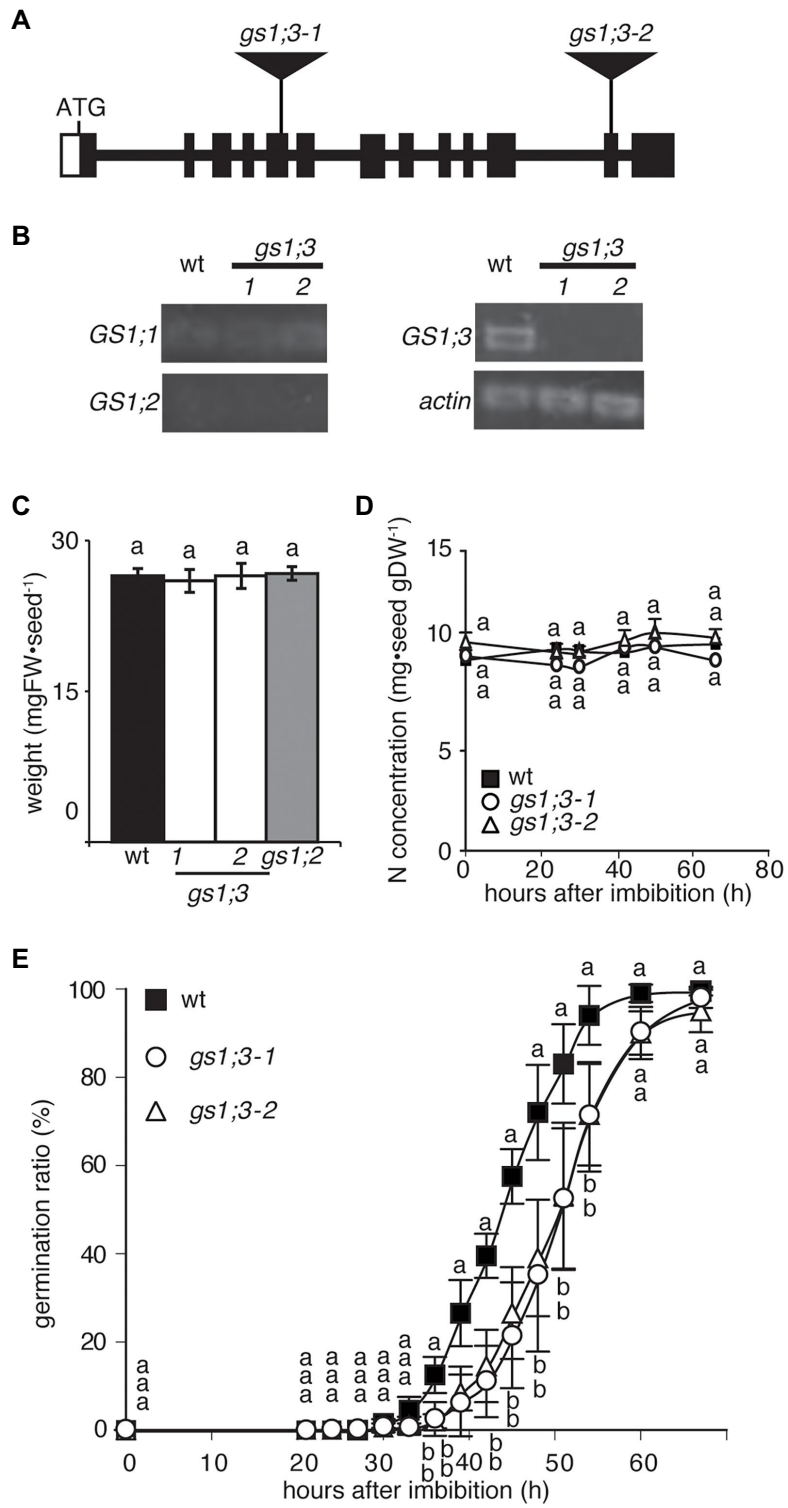
Loss of function of GS1;3 retarded seed germination in rice. **(A)** Schematic representation of the positions of *Tos17* insertions in *gs1;3-1* and *gs1;3-2* mutants. Filled box indicates exon, and line indicates intron. Opened box indicates untranslated region. **(B)** Semi-quantitative RT-PCR analysis (30 cycles) of *GS1* isoforms and *actin* in mature WT and *GS1;3* mutant seeds. **(C)** Weight and **(D)** N concentration of mature WT, *GS1;2* and *GS1;3* mutant seeds. Each data point represents mean ± SD (*n* = 5–10 seeds). **(E)** Germination time course analysis of WT and GS1;3 mutants. During 20–72 h after imbibition, the ratio of germinated seeds was calculated every 3–6 h. Fifty seeds per line were analyzed during this period, and the experiment was repeated three times (*n* = 3 with 50 seeds each). The data represent the mean of 3 experiments ± SD. Significant differences within each group were determined using one-way analysis of variance (ANOVA) followed by Bonferroni tests and are indicated with different letters (*p* < 0.05).

To investigate the physiological function of GS1;3 in seed germination and seedling growth, a reverse genetics approach was used. Two independent *Tos17* insertion lines of *GS1;3* were identified by a screen of pooled *Tos17* insertion line DNA samples. Figure 2A illustrates the *Tos17* insertion position in the *GS1;3* mutant lines. Semi-quantitative real-time PCR (RT-PCR) analysis 24 h after imbibition revealed that *GS1;3* was not expressed in the mutant lines, but in the WT (Figure 2B). By contrast, *GS1;1* and *GS1;2* expressions were detected neither in WT seeds nor in mutant seeds (Figure 2B). *Actin* was expressed in all samples tested (Figure 2B).

Based on the aleurone layer and endosperm specific expression, the expected function of GS1;3 during seed germination and grain quality was analyzed in *GS1;3* mutant and WT seeds. Neither the loss of function of *GS1;3* nor the loss of function of GS1;2 in mutant seeds affected the weight of individual mature seeds, based on seeds selected against a salt water density selection (Figure 2C). The N concentration of mutant and WT seeds did not change significantly during the 72 h observation after imbibition (Figure 2D). Though the weight and N concentration of the *GS1;3* mutant seeds were not changed at 0 h, they took significantly longer to germinate compared to WT seeds (Figure 2E). It took 51 h until 50% of *GS1;3* mutant seeds germinated, whereas 50% of WT seeds germinated in 45 h. Thus, the germination of *GS1;3* mutant seeds was delayed by 6 h compared with WT seeds (Figure 2E).

By contrast, the loss of function of GS1;2 did not affect seed germination (Supplementary Figure S1). Together the data suggest that GS1;3 is involved in the germination process itself.

### *GS1:3* Mutant Lines Showed Altered Amino Acid Levels During Germination

**Figure 3:**
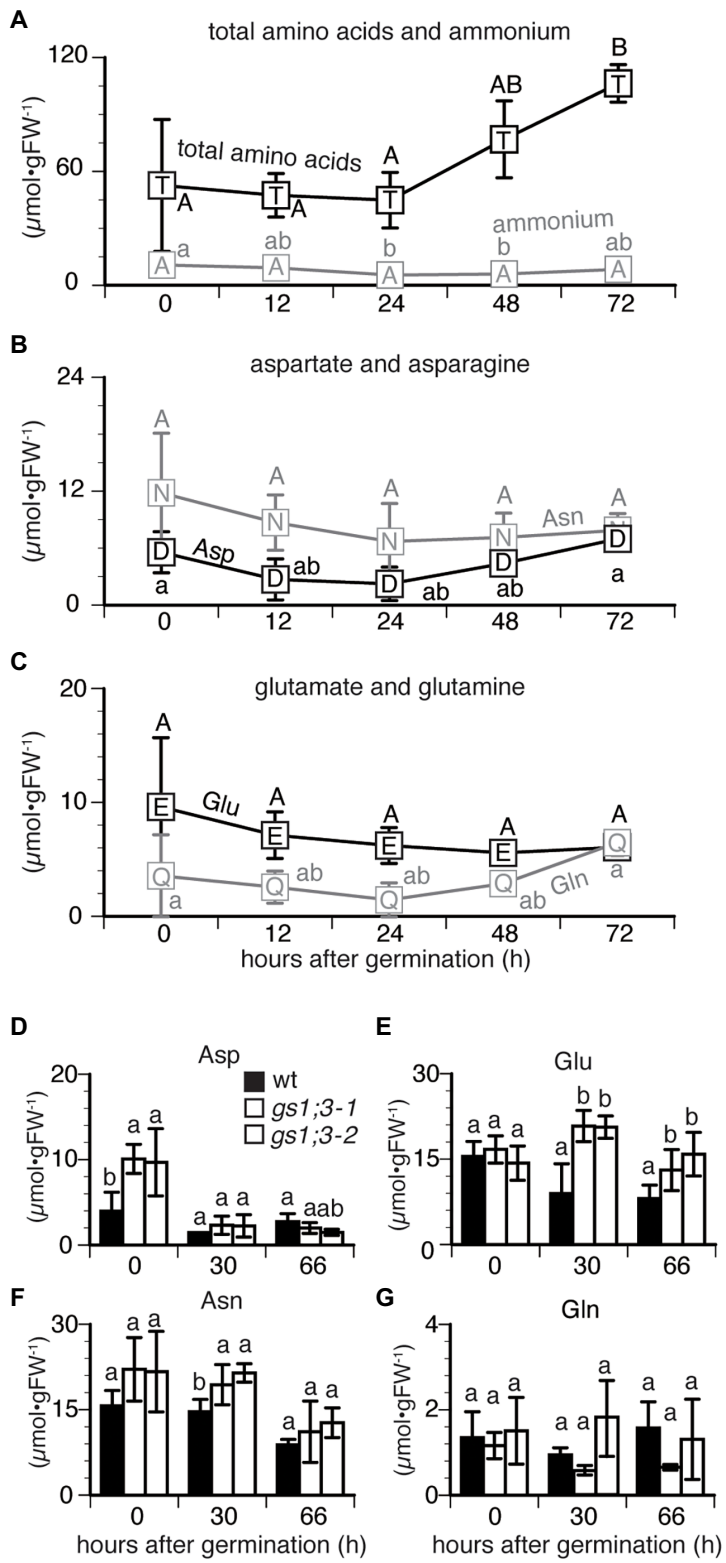
Changes in the concentration of free amino acids and ammonium in germinating rice seeds. Concentrations of **(A)** total amino acids and ammonium and **(B,C)** four selected amino acids in Nipponbare (WT) seeds sampled at 0, 12, 24, 48, and 72 h after imbibition. Data represent mean ± SD (*n* = 3–5). Mature seeds of WT and *GS1;3* mutants harvested at 0, 30, and 66 h after imbibition **(D–G)**. Data represent mean ± SD (*n* = 5). Significant differences within each group **A–C**, changes of each compound in different time and **D–G**, changes of compound at certain time point in different genotypes) were determined using one-way analysis of variance (ANOVA) followed by Bonferroni tests and are indicated with different letters (*p* < 0.05).

For further analysis of the GS1;3 role in germination a time course measurement of ammonium and amino acids was conducted during seed germination. To get an overview of the time-dependent changes, mature WT seeds were analyzed first (Figures 3A–C). In WT seeds, the total concentration of free amino acids started to increase 24 h after imbibition and doubled at 72 h (Figure 3A). The time course did not show significant differences in the concentrations of aspartate (Asp), glutamine (Gln), asparagine (Asn), and glutamate (Glu) over time (Figures 3B,C). However, the concentration of free ammonium slightly decreased from 24 to 48 h after imbibition and increased at 72 h (Figure 3A).

To analyze the GS1;3 specific functions, WT and *GS1;3* mutant lines were analyzed at three time points (Figures 3D–G). The concentration of aspartate (Asp) in *GS1;3* mutant seeds was significantly higher than that in WT seeds at the start of imbibition but sharply decreased after 30 h, reaching a similar concentration level compared to WT seeds (Figure 3D). Initially, the concentration of glutamate (Glu) was comparable in WT and mutant seeds (Figure 3E). However, while the Glu concentration decreased in WT 30 and 66 h after imbibition, it increased temporally at 30 h and decreased again to the initial level at 66 h for *GS1;3* mutant lines (Figure 3E). Asparagine (Asn) showed only a temporal increase in the GS1;3 mutant lines at 30 h, compared to WT, while both WT and mutant plants showed a decrease in Asn at 66 h (Figure 3F). Glutamine (Gln) showed no marked changes in concentration during germination (Figures 3D,G). The data indicate on one hand that the storage components in mature grains of the *gs1;3* mutants might be different (higher Asp at 0 h), which leads to a possible role of GS1;3 in seed ripening. On the other hand, the higher levels of Glu at 30 and 66 h in the mutant seeds indicate involvement in the GS/GOGAT cycle during germination.

Interestingly, though the germination speed was altered in *gs1;3* knockout mutants, no significant differences between WT and *GS1;3* mutants were detected in the plant hormone level in germinating seeds at 24 h after imbibition (Supplementary Figure S2).

### Delayed Seed Storage Use and Slower Seedling Development of *GS1;3* Mutants

To evaluate the GS1;3 loss-of-function effects during germination, *GS1* expression was measured after 10 days of growth under different N regimes. Among the three *GS1* genes, *GS1;1* was expressed at high levels in roots and low levels in shoots, *GS1;2* was mainly expressed in roots, and *GS1;3* was barely expressed in seedlings in WT plants (Supplementary Figure S3). *Actin* was expressed under all conditions (Supplementary Figure S3). None of the genes of GS isoforms showed a differential expression under 5 or 1,000 μM ammonium supply, suggesting that transcriptional regulation by ammonium plays no role in the gene regulation of GS isoforms in the analyzed time-scale.

**Figure 4:**
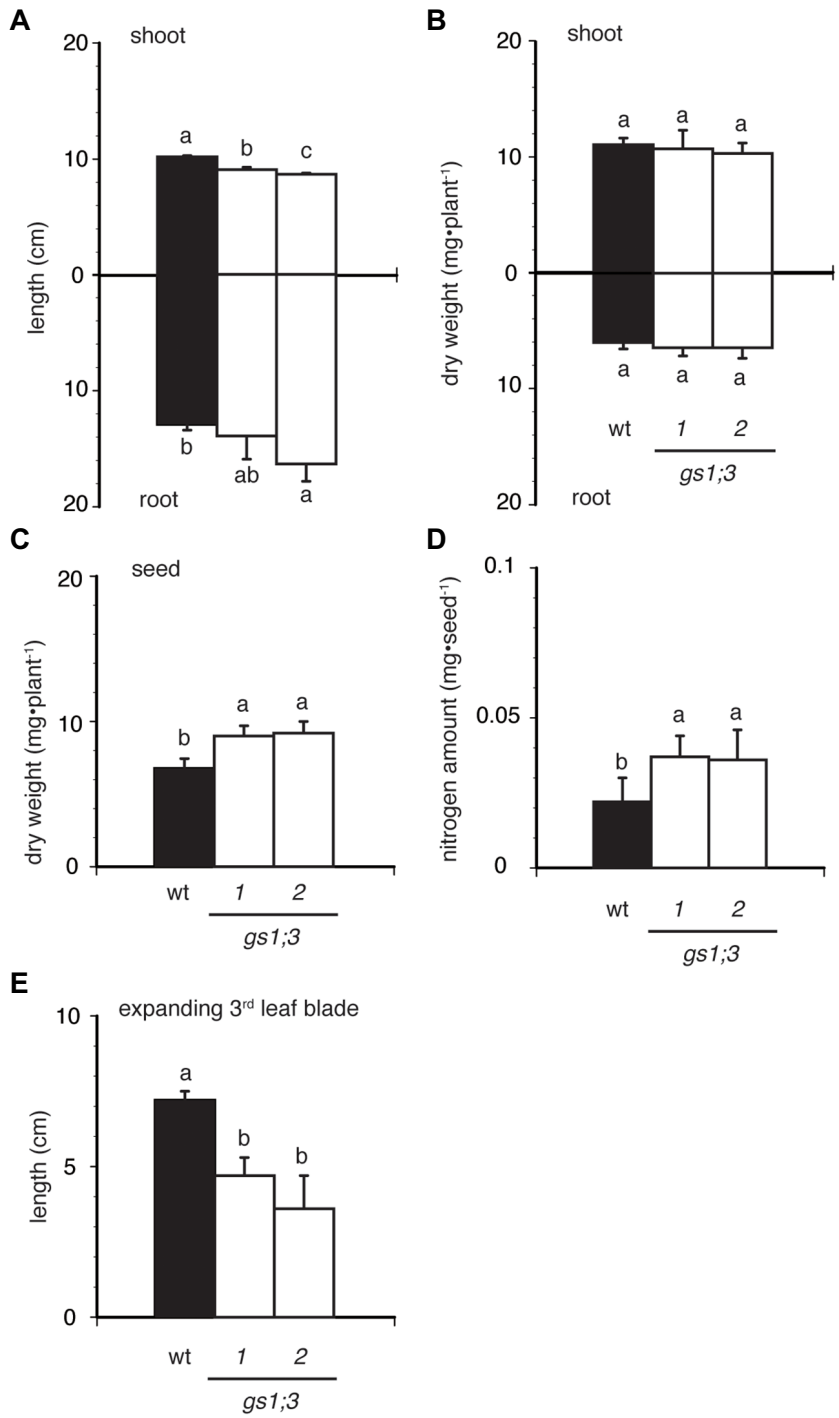
Seedling growth of WT and GS1;3 mutants in water. Seedling growth of WT and GS1;3 mutant plants cultured for 21 days in water. **(A)** root and shoot length, **(B)** root and shoot dry weight, **(C)** seed dry weight, **(D)** seed nitrogen amount after 21 days, and **(E)** third leaf blade length. Data represent mean ± SD (*n* = 5). Significant differences within each group were determined using one-way analysis of variance (ANOVA) followed by Bonferroni tests and are indicated with different letters (*p* < 0.05).

The loss of function of GS1;3 resulted in slight growth retardation in seedlings, when grown in water for 21 days (Figures 4A–E). The two *GS1;3* mutants showed a significant decrease in shoot length, but only one line showed an increased root length compared to WT (Figure 4A). Compared with WT, the root and shoot weight was in both *GS1;3* mutant lines not significantly different (Figure 4B). However, seeds of *gs1;3-1* and *gs1;3-2* were significantly heavier than those of WT 21 days after imbibition (Figure 4C), and the nitrogen amount in the seeds remained higher in the mutants (Figure 4D). Furthermore, the size of expanding 3^rd^ leaf blades (Figure 4E) in GS1;3 mutants was significantly lower compared to WT plants.

This suggests a decreased use of N in the grains during the growth of mutant seedlings since the mature seed weight and N concentration were the same for WT and *GS1;3* mutants before imbibition (Figures 2C,D) while the seeds after 21 days of imbibition retained a higher weight and nitrogen content in *GS1;3* mutants compared to WT plants. Taken together, the lack of GS1;3 resulted in the delayed germination, which led to retardation of the seedling growth. This could be due to limited ability to use nitrogen source in the seed.

### GS1;3 Did Not Influence the Yield of Rice Grown in a Paddy Field

**Figure 5:**
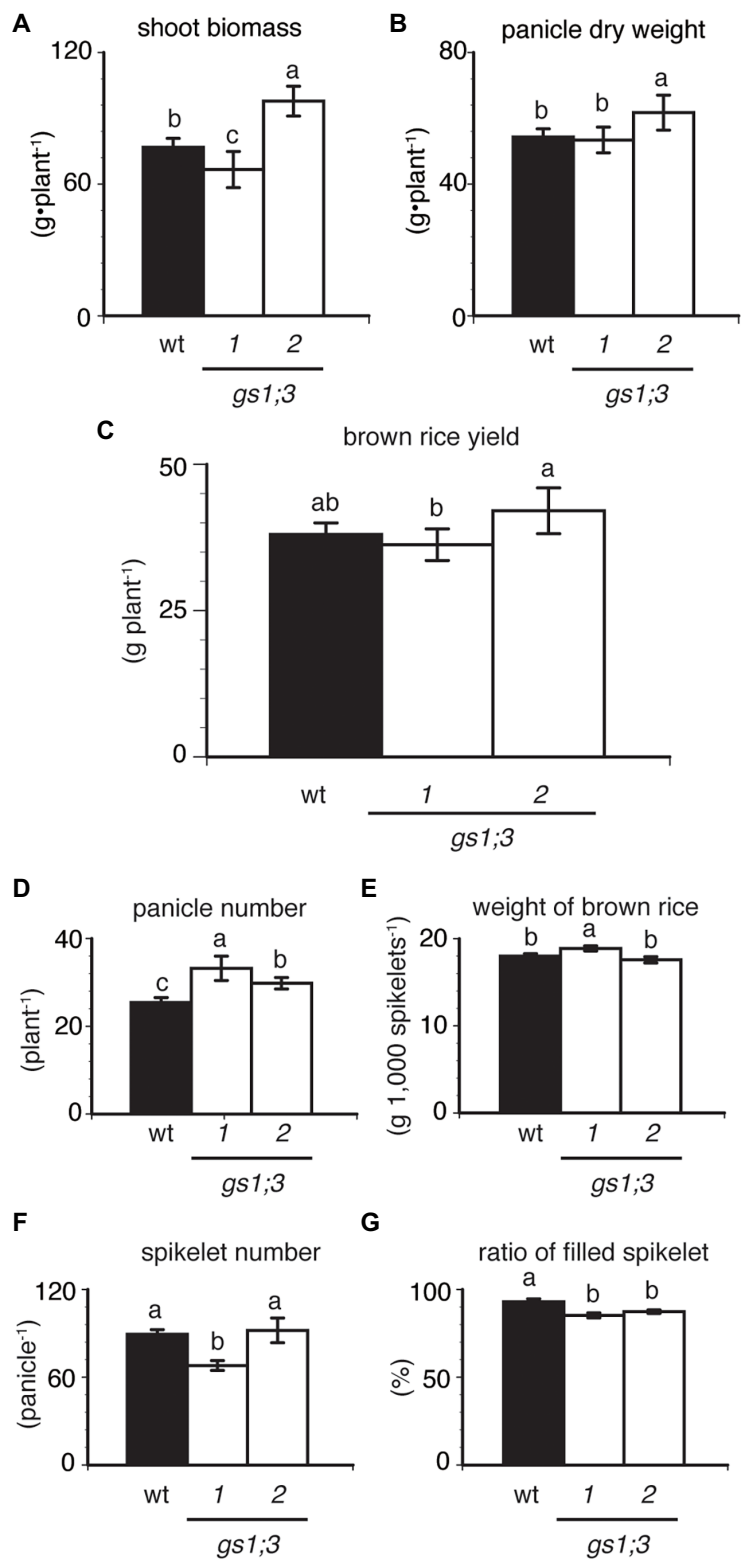
Productivity of GS1;3 mutants grown in the paddy field. **(A)** Shoot biomass, **(B)** panicle dry weight, **(C)** brown rice yield (yield of dehusked, filled spikelets), **(D)** total panicle number, **(E)** weight of brown rice, **(F)** spikelet number per panicle, and **(G)** the ratio of filled spikelets of Nipponbare (WT; closed column) and *GS1;3* mutant (opened columns) plants harvested after 174 days at full maturity are shown. Data represent mean ± SD (*n* = 5). Significant differences within each group were determined using one-way analysis of variance (ANOVA) followed by Bonferroni tests and are indicated with different letters (*p* < 0.05).

While the involvement of GS1;3 was confirmed for the germination and the seedling growth under nitrogen-limited conditions, the question remained if the loss of GS1;3 also influences plant growth in a paddy field with normal nitrogen supply. We analyzed the growth and yield of *GS1;3* mutant and WT plants in a paddy field in Kashimadai, Osaki-shi, Miyagi, Japan. Figure 5 illustrates the biomass and yield of mutant and WT rice at harvest. The shoot biomass of one *GS1;3* mutant plant was heavier, whereas that of the other mutant plant was lighter than the WT (Figure 5A). Neither panicle dry weight (Figure 5B) nor yield (Figure 5C) was the same in *GS1;3* mutant plants. The loss of GS1;3 functions significantly decreased the ratio of filled spikelets (%) by 6–8% compared to WT (Figure 5G). The panicle number was increased by 17–30% in both *GS1;3* mutant plants compared to WT (Figure 5D), whereas the weight of dehusked, filled spikelets (brown rice) and spikelet number per panicle were not the same in *GS1;3* mutant plants (Figures 5E,F). The different trend in several parameters like the shoot biomass and the weight of brown rice suggests that either an unspecified *Tos17* insertion or low-level *GS1;3* transcripts might influence these factors. Only when both lines showed a trend in the same direction the phenotype was regarded as GS1;3 specific. Overall, the field analysis indicated no impact of the loss of function of GS1;3 on yield, but a significant difference in grain filling and panicle number.

### *GS1;3* Mutants Decreased Yield Under N Deficient Conditions

While the analysis of *GS1;3* mutants in paddy led to the identification of its involvement in grain filling, the nitrogen conditions in a paddy are hard to control. A reverse genetics study in maize demonstrated the impact of Gln1_3 and Gln1_4 on N translocation (Martin et al., 2006), and in order to investigate the physiological role of GS1;3 in N translocation, we analyzed the mutants grown with supply of the controlled N source in hydroponic culture. The growth and yield of *GS1;3* mutants and WT plants were compared at harvest when grown with 0.5 mM NH_4_Cl as the sole N source.

**Figure 6:**
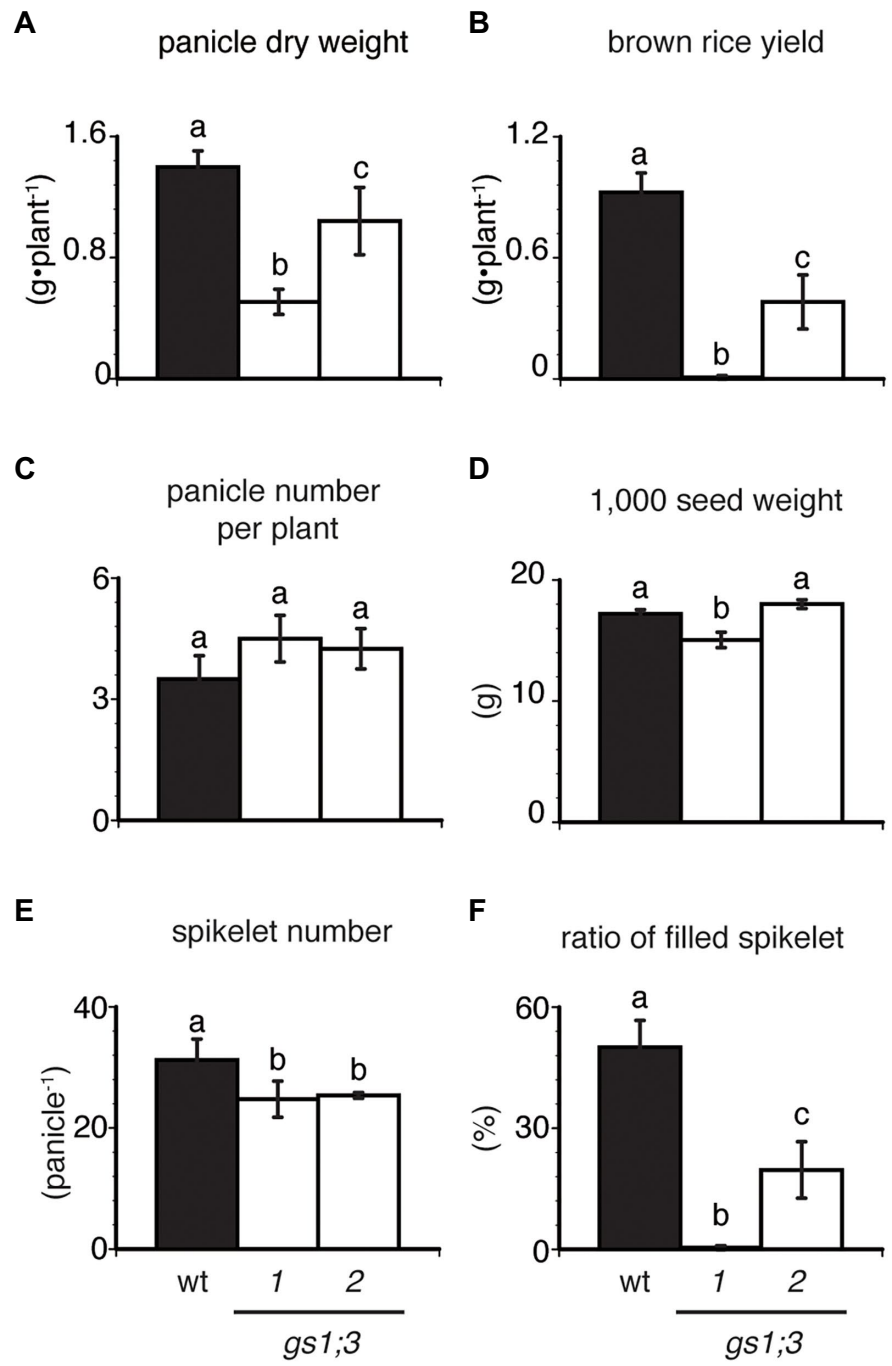
Productivity and growth of GS1;3 mutants in hydroponic culture. **(A)** panicle dry weight, **(B)** brown rice yield, **(C)** panicle number per plant, **(D)** 1,000 seed weight, **(E)** spikelet number per panicle, and **(F)** ratio of filled spikelets of WT and *GS1;3* mutant plants grown in hydroponic culture with 0.5 mM NH_4_^+^ are shown. All samples were harvested 61 days after heading. Data represent mean ± SD (*n* = 5). Significant differences within each group were determined using one-way analysis of variance (ANOVA) followed by Bonferroni tests and are indicated with different letters (*p* < 0.05).

The panicle dry weight of individual *GS1;3* mutant plants was reduced by 26–64% at harvest (Figure 6A). The yield of *GS1;3* mutants was more than 60% lower than that of the WT (Figure 6B). To identify what led to the yield decrease, a yield component analysis was performed. As the cause of the mutant yield decrease, a 20% reduction in spikelet number per panicle (Figure 6E) and > 30% reduction in the ratio of filled spikelets (Figure 6F) was identified. However, the panicle number was not significantly different (Figure 6C), and the 1,000-spikelet weight (Figure 6D) showed only a decrease for one of the two *GS1;3* mutant lines compared to WT. This indicates that GS1;3 is mainly involved in grain filling during ripening.

### GS1;3 Influences N Translocation and Panicle Development Under N Deficient Conditions

**Figure 7:**
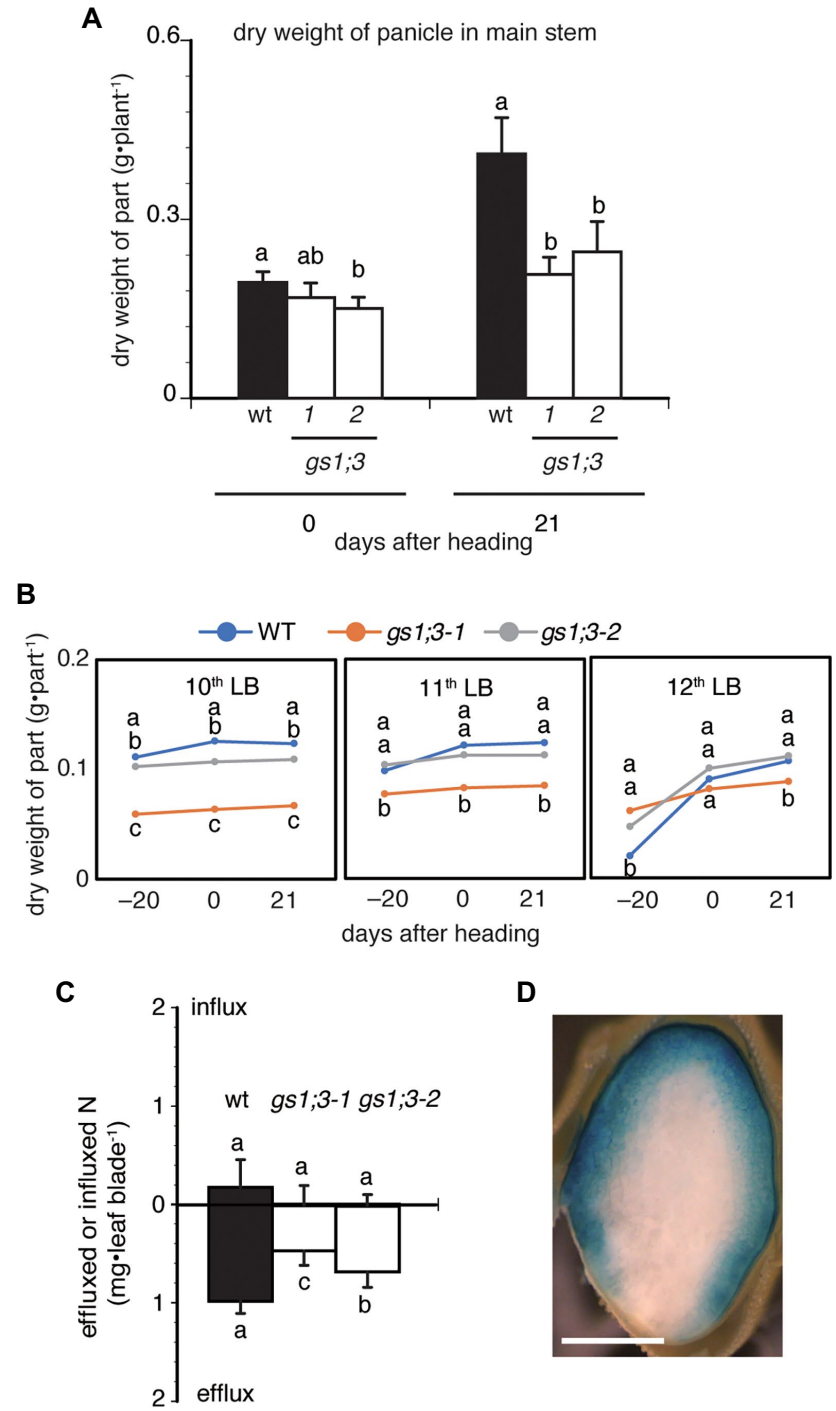
Influx and efflux of nitrogen in the 12th leaf blade on the main stem and GS1;3 promoter activity in ripening grains. **(A)** Dry weight of main stem panicles, and **(B)** dry weights of the 10th to 12th leaf blades of WT and GS1;3 mutant plants grown in hydroponic culture with 0.5 mM NH_4_^+^ are shown. For **(A,B)**, plant organs were harvested 19 days before heading, at heading, 20 days after heading. **(C)** The influx and efflux of nitrogen in WT and *GS1;3* mutant plants in the 12th leaf blades at 61 days after heading. Plants were grown in hydroponic culture and were supplied with ^15^N-labeled NH_4_Cl for 5 days from 19 days before heading onwards. Significant differences within each group were determined using one-way ANOVA followed by Bonferroni tests and are indicated with different letters (*p* < 0.05). Data represent mean ± SD (*n* = 8, for wt, *gs1;3-1*, and *gs1;3-2*; **D**) *GS1;3* promoter-driven GUS activity in ripening rice grain. Transgenic plants expressing *GS1;3pro::GUS* were grown in the greenhouse on fully fertilized soil. Ripening grains were harvested at 35 days after heading and used for GUS staining. The transverse section is shown. Bar indicates 1 mm.

To further analyze the cause of the reduced yield, the panicle weight of the main stem was measured at heading and 21 days after heading. One of two *GS1;3* mutant lines showed a significant decrease in the dry weight of the main stem panicles at the heading date, and both lines showed a decrease to around 50% of WT main stem panicle weight 21 days after heading (Figure 7A). The much lower weight of the mutant main stem panicles 21 days after heading is based on the significant increase of the WT main stem panicle and the marginal increase of the mutant main stem panicles compared to the heading date (Figure 7A). To confirm that GS1;3 is involved in the N translocation, ^15^N-labeled NH_4_Cl was supplied to trace the N movement. Plants were labeled with ^15^N at 19 days before heading when the 12th leaf was emerging. The 12th leaf blade was chosen as a proxy to determine the N translocation rate since the growth rate was minimal between the heading date and 21 days after (Figure 7B). The influx and efflux of N in the 12th leaf blade over the time course, ranging from 19 days before heading to 61 days after heading show that in WT, the N efflux was 5-fold higher than the N influx (Figure 7C). In *GS1;3* mutants, the N efflux showed a significant reduction (21–38%) in the 12th leaf over time compared to the WT; however, no significant difference was detected in the N influx between WT and *GS1;3* mutants (Figure 7C). In addition to the nitrogen translocation, the analysis of a *GS1;3* promoter-driven GUS activity in a ripening grain at 35 days after heading revealed the localization of the promoter activity in the dorsal vascular bundles and aleurone layer (Figure 7D). These data support a role of GS1;3 in N translocation to seeds during the grain filling stage.

## Discussion

### GS1;3 Function in the Aleurone Layer and Endosperm Promotes Seed Germination in Rice

After imbibition *GS1;3* showed a specific expression in the aleurone layer followed by a delayed expression in the endosperm, while *GS1;1* and *GS1;2* were expressed in the emerging plants, and lowly in the germinating seeds (Figure 1). The expression pattern of rice *GS1;3* is comparable to the barley *GS1;3* orthologue, *HvGS1_3*, which also showed an expression in the endosperm and was suggested to be important for seed maturation and germination, with a suggested role in the assimilation of ammonium from protein degradation in senescing leaves and/or seed reserves (Goodall et al., 2013). The localization of both, rice and barley GS1;3 isoforms are slightly different from wheat GS1;3, which showed an expression in the transfer cells, indicating a function in translocation similar to GS1;2 in barley and rice (Tabuchi et al., 2007; Goodall et al., 2013; Wei et al., 2021).

The delayed germination of the *gs1;3* mutants (Figure 2) is likely based on the involvement of GS1;3 in storage component mobilization, that is, the assimilation of free ammonium derived from proteolysis. The limited N assimilation over time due to lack of the GS1;3 function in *gs1;3* mutants resulted in the increase in the remaining nitrogen concentration in form of glutamate compared to WT (Figure 3E). The free ammonium level was unchanged in the GS1;3 mutants (data not shown) compared to WT, indicating that the conversion of storage protein to free ammonium is feedback regulated by the glutamate level. This supports the role of GS1;3 suggested by Goodall et al. (2013).

In addition to that function, it is likely that GS1;3 is important for the energy status in the germinating seed. The germinating seed is heterotroph, and the supply of oxygen is limited due to the dense seed structure as well as to waterlogged conditions in the case of rice seeds. Amino acids can serve as energy donors through their catabolism in the TCA cycle (Angelovici et al., 2011; Araújo et al., 2011; Kirma et al., 2012), and the aspartate family pathway plays a crucial role under energy shortage (Galili, 2011; Credali et al., 2013). When we analyze the amino acid data (Figure 3), we see an accumulation of Asn after 30 h, and an accumulation of Glu after 30 and 66 h in the *GS1;3* mutant seeds. The role of Asn as an energy donor starts from its conversion to aspartate, the precursor of the branched Asn family pathway (Mini-review by Galili et al., 2014). Considering that Asp can be converted by the Asp aminotransferase to Glu, a substrate of GS1;3, and that gln, a product of GS1;3, is an important precursor to produce Asn *via* the Asn synthetase (Gaufichon et al., 2016), we can assume a detrimental effect in the initial steps of the energy donor function of Asn in the GS1;3 mutant lines. Since no energy is needed for the action of the Asp aminotransferase, an equilibrium between the glutamate and aspartate concentration is likely. With the decreased function of GS1;3, we see the increased levels of glutamate after 30 and 66 h. The accumulation of Asn could be a result of the product accumulation (aspartate) which could decrease the asparaginase activity. Since this blocks the initial step in this pathway, the energy production is reduced. Though amino acids are precursors of phytohormones, the phytohormone level is unchanged in the *GS1;3* mutant plants (Supplementary Figure S2).

The role of rice GS1;3 in germinating seeds is therefore to assimilate ammonium derived from protein catabolism in the aleurone layer and the endosperm, which is linked to the energy status that is needed to maintain the storage protein proteolysis for seedling growth.

### Nitrogen Limitation Enhances *GS1;3* Importance for Seed Ripening in Rice

Under normal field conditions, the only remarkable phenotype of the *GS1;3* mutants was a significant reduction in the ratio of filled spikelets (Figure 5G). This suggests that GS1;3 is not important for vegetative growth, besides the initial involvement in seed germination, which is consistent with former studies about major GS1 isoforms in rice (Tabuchi et al., 2005, 2007). However, the reduction in the ratio of filled spikelets suggested involvement in seed ripening in addition to germination.

Since N supply is one of the major factors, which influence grain filling and yield (Uribelarrea et al., 2004), and *GS1;3* is expressed in ripening spikelets (Tabuchi et al., 2005), the *GS1;3* mutants were analyzed under nitrogen-limited conditions.

The reduction of nitrogen supply markedly reduced the yield of *GS1;3* mutant plants, mainly based on the reduction of the ratio of filled spikelets, leading to a decrease in panicle dry weight and brown rice, with a smaller but still significant decrease in the spikelet number (Figure 6).

The localization of rice GS1;3 in spikelets is in the endosperm and the aleurone layer (Figure 7D). While the effect of different GS1 isoforms on yield is well documented, most articles focus on isoforms that are important during vegetative growth, like Gln1_3 and Gln1_4, which exert their function due to their localization in the mesophyll and bundle sheath cells of maize leaves (Martin et al., 2006). The only known GS1 isoforms with predominant localization in seeds are GS1;3 in wheat (Wei et al., 2021), and GS1;3 in barley (Goodall et al., 2013), *GLN1;5* in Arabidopsis (Schmid et al., 2005; Winter et al., 2007; Bassel et al., 2008; Le et al., 2010), and GS1;3 in rice (this study).

Considering the specific localization in spikelets and seeds, likely explanations for the phenotype are similar to the germinating seeds, namely, the involvement in the GS/GOGAT cycle for the build-up of the storage proteins, as well as its involvement in the energy metabolism.

As reviewed by Galili et al., 2014, monocotyledonous plants do not generate energy through photosynthesis in seeds, leaving again the asparagine pathway as a possible root for energy supply. Indeed, the Asp concentration in *GS1;3* mutant line seeds was significantly higher compared to WT upon imbibition (Figure 3), suggesting that the production of energy is affected by the lack of GS1;3, due to the disturbance of the GS/GOGAT cycle in the endosperm/aleurone layer. Further support for this hypothesis is given in Figure 7A, which showed that the developing main stem panicle nearly doubled its weight in WT seeds 21 days after heading, while *GS1;3* mutant seeds just slightly increased. With the production of storage components during heading, the spikelets get thicker, making them more and more impenetrable to oxygen, which is increasing the need for alternative energy sources, which cannot be supplied anymore in the case of GS1;3 seeds.

Since the plant weight in the *GS1;3* mutant lines was not affected, they do not have a nitrogen limitation *per se*. When the 12th leave was used as a proxy for nitrogen translocation (Figures 7B,C), it was shown that the nitrogen efflux from the leave was reduced, which can be explained by a reduction in the sink strength (reduced ratio of filled spikelets) caused by the lack of GS1;3.

In conclusion, the GS1;3 involvement in seed germination and its influence on spikelet filling, which is more pronounced under nitrogen limitation, is based on its involvement in the assimilation of free ammonium in the aleurone layer and the endosperm during storage protein biosynthesis and degradation. It furthermore seems to be involved in the energy provision that fuels these processes. Future research will focus on the contribution of GS1;3 to the energy metabolism in non-photosynthetic tissues.

### Outlook

The importance of GS1;3 under nitrogen limitation makes it a possible target for improving crop productivity. A possible approach might be an overexpression of GS1;3, which has the potential to improve yield specifically under nitrogen-limited conditions. Successful examples are the overexpression of GS1;1 and GS1;2, that led to an improved tolerance to abiotic stress in rice (James et al., 2018), and the overexpression of GS1-1, which improved the nitrogen utilization efficiency in barley (Gao et al., 2019). Considering the assumed role of GS1;3 in the energy metabolism a positive effect might occur also in developing seeds without nitrogen limitation.

## Data Availability Statement

The original contributions presented in the study are included in the article/Supplementary Material, further inquiries can be directed to the corresponding author.

## Author Contributions

MT-K, KI, TH, TY, and SK contributed to conception and design of the study. MT-K, TU-O, EM, and HN performed the promoter analysis. YH, KS, and TF performed the seed germination analysis. MT-K and YH performed the amino acid measurement. TF, MT-K, YH, HN, KS, YS, and SK performed the growth analysis. MK, HS, HN, and KI performed the hormone analysis. TF, MT-K, YS, and AM isolated the *Tos17* insertion lines. TF, MB, and SK performed the tracing analysis. MB and SK wrote the first draft of the manuscript. All authors contributed to the article and approved the submitted version.

## Funding

Japan Advanced Plant Research Network supported by JSPS was also acknowledged for the use of Elemental Analyzer. JSPS KAKENHI Grant Numbers, 21688006 and 26450073 to SK and 22119003 to TY supported this work. TH was supported by a Grant-in-Aid for Scientific Research from the Ministry of Education, Culture, Sports, Science, and Technology of Japan (KIBAN B; 17H03780). This study was supported by the Network of Centers of Carbon Dioxide Resource Studies in Plants (NC-CARP).

## Conflict of Interest

The authors declare that the research was conducted in the absence of any commercial or financial relationships that could be construed as a potential conflict of interest.

## Publisher’s Note

All claims expressed in this article are solely those of the authors and do not necessarily represent those of their affiliated organizations, or those of the publisher, the editors and the reviewers. Any product that may be evaluated in this article, or claim that may be made by its manufacturer, is not guaranteed or endorsed by the publisher.

## Footnotes

^1^ http://tos.nias.affrc.go.jp/
