# Supplementary material for: Cytosolic Glutamine Synthetase GS1;3 Is Involved in Rice Grain Ripening and Germination
Source: Front Plant Sci. 2022 Feb 8;13:835835. doi: 10.3389/fpls.2022.835835 (PMC8861362; doi:10.3389/fpls.2022.835835)
Supplement: Supplementary file 1 [file Table_1.PDF]

Table S1. Primers used in this study

Purpose of use	Gene name	other	Fwd / Bwd	Reported	Sequence(5' to 3')
nested PCR 1		Tos17	Fwd		ACCACTTCAGAGATTGTGTGGTTGC
		Tos17	Bwd		CAGCAACGATGTAGATGGTCAAGC
	GS1;3		Fwd		GACCTCGTTAACCTCGACCT
			Bwd		GTGGTCTCGGCGATCATG
nested PCR 2		Tos17	Fwd		GACAACACCGGAGCTATACAAATCG
		Tos17	Bwd		AGGAGGTTGCTTAGCAGTGAAACG
	GS1;3		Fwd		AAGGTCATCGCCGAGTACAT
			Bwd		GGGTCCATGTTGGACGCCGGCC
Genotyping ND0163	GS1;3		Fwd		GGGTTTTCTTCGGTTCTTCC
			Bwd		TACCTGAGGGCCTGGATAGCCACCTA
Genotyping Ne4721	GS1;3		Fwd		GGACAAGATCGCTCGTCACACTCATC
			Bwd		ATCAATGGCGACGCCTCCTTTACCTT
promoter	GS1;3		Fwd		AAAAAGCAGGCTGGCATCCTCTTCTTCCCGCTTGCTT
			Bwd		AGAAAGCTGGGTTGTACTCGGCGATGACCTTGTCCGT
promoter flanking attB			Fwd		ACAAGTTTGTACAAAAAAGCAGGCT
			Bwd		ACCACTTTGTACAAGAAAGCTGGGT
qPCR	GS1;1		Fwd	Ishiyama et al., 2004	CATCAACCTTAACCTCTCAGACTCCACT
			Bwd		ACTTCAGCTGCAACATCAGGGTTGCTA
	GS1;2		Fwd	Tabuchi et al., 2005	AAAGGCGTTCGGCCGCGACATCGTGGAC
			Bwd		CACTTGGTCAGCAGCGGCGATG-CCAACT
	GS1;3		Fwd	Tabuchi et al., 2005	TAAATCGTACGGGCGCGACATCGTTGAT
			Bwd		GACATGATCCCCTGCGGAGACGCCAA
	actin		Fwd	Sonoda et al., 2003	CTTCATAGGAATGGAAGCTGCGGGTA
			Bwd		CGACCACCTTGATCTTCATGCTGCTA
