# Supplementary material for: Cytosolic Glutamine Synthetase GS1;3 Is Involved in Rice Grain Ripening and Germination
Source: Front Plant Sci. 2022 Feb 8;13:835835. doi: 10.3389/fpls.2022.835835 (PMC8861362; doi:10.3389/fpls.2022.835835)
Supplement: Supplementary file 2 [file Presentation_1.PDF]

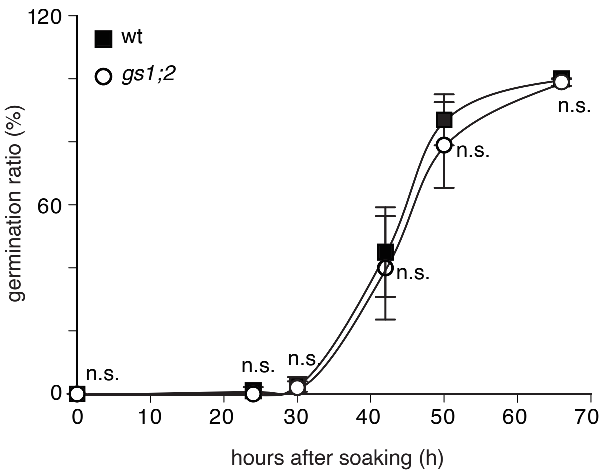

Figure S1. Changes in germination ratio during 72 h after immersion.

Fifty mature seeds were placed on a filter paper moistened with 4.5 mL distilled water in a Petri dish. Seeds were incubated at 30ºC for 72 h. The ratio of germinated seeds was calculated in 6-hour intervals. 50 seeds per line were analyzed during this period, and the experiment was repeated 3 times (n = 3 with 50 seeds each). At 6 h intervals, 200 μL of water was supplemented to compensate for the changes in the water level. No significant differences were observed in the germination ratio between WT and *GS1;2* mutant seeds.
