# Supplementary material for: Cytosolic Glutamine Synthetase GS1;3 Is Involved in Rice Grain Ripening and Germination
Source: Front Plant Sci. 2022 Feb 8;13:835835. doi: 10.3389/fpls.2022.835835 (PMC8861362; doi:10.3389/fpls.2022.835835)
Supplement: Supplementary file 3 [file Presentation_2.PDF]

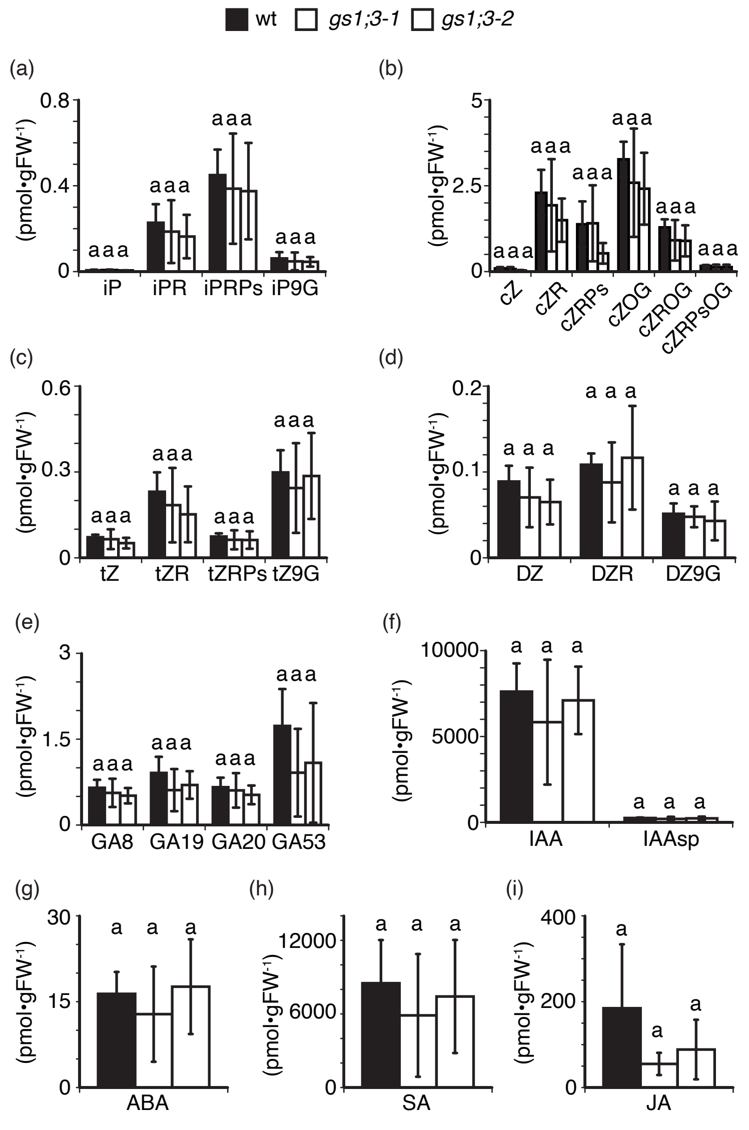

Figure S2. Contents of free cytokinin, gibberellic acid, auxin, abscisic acid, salicylic acid, and jasmonic acid in germinating rice seeds.

Contents of (a) iP cytokinin, (b) cZ cytokinin, (c) tZ cytokinin, (d) DZ cytokinin, (e) gibberellic acid, (f) indole acetate and aspartate-conjugated indole acetate, (g) abscisic acid, (h) salicylic acid, and (i) and jasmonic acid in germinating rice seeds. Data represent mean ± SD (n = 3–4). No significant differences were observed in hormone contents between WT and *GS1;3* mutant seeds.
