# Supplementary material for: Cytosolic Glutamine Synthetase GS1;3 Is Involved in Rice Grain Ripening and Germination
Source: Front Plant Sci. 2022 Feb 8;13:835835. doi: 10.3389/fpls.2022.835835 (PMC8861362; doi:10.3389/fpls.2022.835835)
Supplement: Supplementary file 4 [file Presentation_3.PDF]

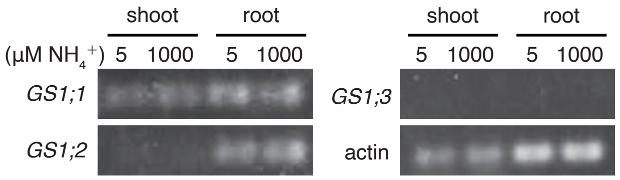

Figure S3. RT-PCR analysis of root RNA from Nipponbare.

Plants were cultured under continuous supply of either 5 μM or 1000 μM ammonium chloride as the sole nitrogen source for 10 d. Gene-specific primers were used for RT-PCR analysis with 35 cycles.
